# Cr-Doped Urchin-Like WO_3_ Hollow Spheres: The Cooperative Modulation of Crystal Growth and Energy-Band Structure for High-Sensitive Acetone Detection

**DOI:** 10.3390/s20123473

**Published:** 2020-06-19

**Authors:** Qiongling Ding, Yanrong Wang, Pengqian Guo, Jianjun Li, Chen Chen, Ting Wang, Kai Sun, Deyan He

**Affiliations:** School of Physical Science and Technology, Lanzhou University, Lanzhou 730000, China; dingql18@lzu.edu.cn (Q.D.); guopq15@lzu.edu.cn (P.G.); jjli19@lzu.edu.cn (J.L.); chench2018@lzu.edu.cn (C.C.); wangting2017@lzu.edu.cn (T.W.); sunk2019@lzu.edu.cn (K.S.); hedy@lzu.edu.cn (D.H.)

**Keywords:** hierarchical nanostructure, Cr-doped WO_3_, urchin-like, acetone sensor, crystal growth

## Abstract

Acetone is a biomarker in the exhaled breath of diabetic patients; sensitive and selective detection of acetone in human exhaled breath plays an important role in noninvasive diagnosis. Tungsten oxide (especially for γ-WO_3_) is a promising material for the detection of breath acetone. It is generally believed that the stable metastable phase of WO_3_ (ε-WO_3_) is the main reason for the improved response to acetone detection. In this work, pure and Cr-doped urchin-like WO_3_ hollow spheres were synthesized by a facile hydrothermal approach. Analyses of the resulting materials via X-ray photoelectron spectroscopy (XPS) and Raman confirmed that they are mainly composed by γ-WO_3_. The gas sensing performances of pure and Cr-doped WO_3_ to acetone were systematically tested. Results show that the sensor based on pure WO_3_ annealed at 450 °C has a high response of 20.32 toward 100 ppm acetone at a working temperature of 250 °C. After doped with Cr, the response was increased 3.5 times higher than the pure WO_3_ sensor. The pure and Cr-doped WO_3_ sensors both exhibit a tiny response to other gases, low detection limits (ppb-level) and an excellent repeatability. The improvement of gas sensing properties could be attributed to an optimized morphology of Cr-doped WO_3_ by regulating the crystal growth and reducing the assembled nanowires’ diameter. The increasing number of oxygen vacancy and the introduction of impurity energy level with trap effect after Cr doping would lead to the wider depletion layer as well as a better gas sensing performance. This work will contribute to the development of new WO_3_ acetone sensors with a novel morphology and will explain the increased response after Cr doping from a new perspective.

## 1. Introduction

With the rapid development of industrial technology, air quality and indoor pollution have resulted in serious health problems [1,2,3]. Diabetes is a metabolic disease caused by insufficient insulin secretion or insulin utilization disorders [4]. The acetone concentration in exhaled breath is 300~900 ppb for healthy human but more than 1800 ppb for diabetic patients [5,6,7,8,9,10]. Therefore, accurate and selective detection of acetone is considered as a promising diagnostic tool for a noninvasive health check [11,12]. Solid-state gas sensors are an important means for breath analysis [13]. Among the various kinds of gas sensors, semiconductor oxide (SMO) based gas sensors are widely studied because of their low cost, real-time monitoring and long service life [13,14,15,16,17]. The conductivity of semiconductor oxide, due to the existence of its band gap, will change when it interacts with the target gas, and various information of the gas can be obtained [18,19,20]. Some metal oxide semiconductors such as WO_3_, SnO_2_, and ZnO have been proven to be used in the detection of acetone [20]. Tungsten oxide (WO_3_) is a typical n-type metal oxide material with a wide band gap of 2.4–2.8 eV at room temperature [21]. WO_3_ is widely used in various fields, such as solar cells [22], supercapacitors [23], photocatalysis [24] and sensors [25,26,27,28,29,30,31,32,33,34], due to its unique electronic properties. In addition, as a kind of a low toxic, stable and low-cost SMO material, WO_3_ can be more readily adopted for practical applications [35]. Commercial WO_3_ materials have very low response to various gases due to their low reactive sites. Therefore, synthesis of hierarchical nanostructures with higher specific surface area and porosity to improve gas sensitivity to a greater extent is still a hotspot of gas sensing research [36], which includes nanospheres [37], nanorods [38], nanoflakes [39], nanoflowers [40] and so on. WO_3_ crystals are generally formed by corner and edge sharing of WO_6_ octahedra, and evolve into the following crystal phase: monoclinic I (γ-WO_3_), monoclinic II (ε-WO_3_), triclinic (δ-WO_3_), orthorhombic (β-WO_3_), hexagonal (h-WO_3_), tetragonal (α-WO_3_), and cubic WO_3_ [41]. The crystal phase of WO_3_ can be changed during annealing and cooling processes, just like other SMOs.

In these crystal phases, γ-WO_3_ and ε-WO_3_ have been widely investigated for the detection of acetone due to their higher response to acetone than other gases [42]. However, ε-WO_3_ is a metastable phase which is not stable at room temperature [43]. Recently, it has been reported that adding Si, Cr or C element in the lattice of γ-WO_3_ can stabilize the phase and thus improve the response for acetone [44,45,46]. For instance, Shen et al. reported porous C-doped WO_3_ hollow spheres synthesized by a carbon-sphere template method, which is proved to be a mixture of ε and γ-phase. The results show that these sensors have a higher sensitivity to acetone than pure WO_3_ materials [46]. Liu et al. synthesized Co-doped WO_3_ flower-like nanostructures assembled with nanoplates (FNPs) by a one-step hydrothermal route. The response of 0.6 at% Co-doped WO_3_ FNPs based sensor to 300 ppm acetone gas can reach 151, while ε-WO_3_ doesn’t exist [47]. Moreover, there are also other acetone sensors based on grapheme [48], composites [49], organic materials [50,51], and biological materials [52] that have been studied and reported.

In the present work, the pure and Cr-doped urchin-like WO_3_ hollow spheres were successfully synthesized via a simple hydrothermal method, followed by annealing at different temperatures. The growth process of urchin-like WO_3_ hollow spheres was explored. Structural and morphological characterization revealed that the resulting materials are mainly composed of γ-WO_3_ and the morphology can be optimized by Cr doping. The gas sensing performances to acetone vapor with the change of annealing temperature and Cr doping amount were systematically investigated. The results show that 450 °C is the optimal annealing temperature of the material, because of the relatively high specific surface area and oxygen vacancy. When the doping amount is 100 mg, the sensor exhibits the best response, which is 3.5 times higher than the pure WO_3_ sensor. Since ε-phase WO_3_ does not form in this case, the increased response to acetone after Cr doping is explored here from a new perspective. The approach and results proposed in this work may contribute to the realization of more sensitive acetone detection.

## 2. Experimental

### 2.1. Materials and Synthesis

All chemicals used in the experiments are analytical reagent grade without further treatment. Pure WO_3_ was synthesized by a simple hydrothermal method and followed by a calcination process. In a typical experiment, 1 g WCl_6_ was dissolved in the solution containing 50 mL of ethanol and 4 mL of ethylene glycol under continuous stirring. After 30 min, 200 µL of 12 M HCl was added dropwise to the clear solution and kept stirring for a while. Then, the above mixture solution was transferred into a 100 mL Teflon-lined stainless-steel autoclave and kept at 180 °C for 12 h. After naturally cooling down to room temperature, the resulting precipitates were collected by centrifugation and washed several times with deionized water and absolute ethanol. Then, the predecessor was placed in a drying oven at 60 °C for 24 h. After that, the as-prepared product was annealed in a muffle furnace which was heated up (2 °C min^−1^) to 400, 450, 500 and 550 °C and maintained for 2 h, respectively. The corresponding products were labeled as W-400, W-450, W-500 and W-550. 

Cr-doped WO_3_ was obtained under the same experimental conditions, except that different amounts of Cr(acac)_2_ (10, 50, 100 and 150 mg) were added in the precursor; the corresponding calculated molar ratios of Cr: W were 1.1:100, 5.6:100, 11.4:100 and 17.0:100, respectively. After annealing the products in air at 450 °C for 2 h, the Cr-doped WO_3_ sample was obtained. The corresponding products were marked as WCr-0mg, WCr-10mg, WCr-50mg, WCr-100mg, and WCr-150mg, respectively.

### 2.2. Characterization

Thermal gravimetric analysis (TGA, Du Pont Instrument 1090B) was carried out in air with a heating rate of 5 °C min^−1^. The structures and morphologies of the samples were characterized by field-emission scanning electron microscopy (FE-SEM, Hitachi S-4800), high-resolution transmission electron microscopy (HR-TEM, FEI Tecnai G2 F30) equipped with scanning transmission electron microscopy (STEM) and energy dispersive X-ray spectroscopy (EDX). The crystalline phases and composition of the samples were analyzed by X-ray powder diffraction (XRD, Rigaku RINT2400 with a Cu Kα radiation) and micro-Raman spectrometer (Jobin–Yvon Horiba HR800 with an excitation wavelength of 532 nm). X-ray photoelectron spectroscopy (XPS) analysis was carried out using a Kratos Axis Ultra DLD instrument with Al Kα probe beam. Ultraviolet photoelectron spectroscopy (UPS) equipped with He-Iα 21.22 eV UV light was used to measure the valence band. 

### 2.3. Sensor Fabrication and Measurement

**Figure 1:**
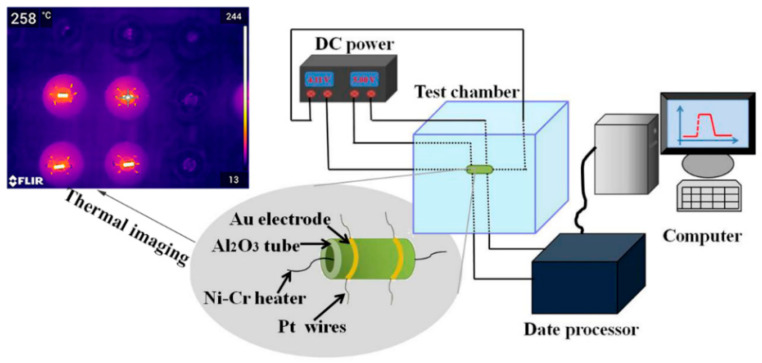
Schematic illustration of the experimental platform and thermal image of the device.

The obtained samples were mixed with a small amount of binder (Ethyl cellulose: Terpineol = 1:9) and ground together in an agate mortar to form a paste. Then it was uniformly coated onto the surfaces of Al_2_O_3_ ceramic tube with a pair of Au electrodes and four Pt wires. After dried in air, a calcinations step at 400 °C for 2 h was performed to completely remove the organic binder and make the material stick tightly on a ceramic tube. A Ni–Cr resistor was inserted into the ceramic tube for controlling the working temperature by adjusting the voltage. The ceramic tube was welded on a special pedestal with six poles to measure the sensing performance [53]. Before testing, the sensor was aged for 24 h on the aging table (TS-64B) to stabilize the resistance. The sensor was installed into a test chamber. Ambient humidity and temperature were maintained at 40% and 30 °C during the test, respectively. Schematic illustration of the experimental platform and a thermal image of the heated ceramic tube are shown in Figure 1. The sensor response was defined as S = R_air_/R_gas_, where R_air_ and R_gas_ are the sensor resistances measured in air and in analyte gas atmosphere, respectively [54].

## 3. Results and Discussion

### 3.1. Growth Process

**Figure 2:**
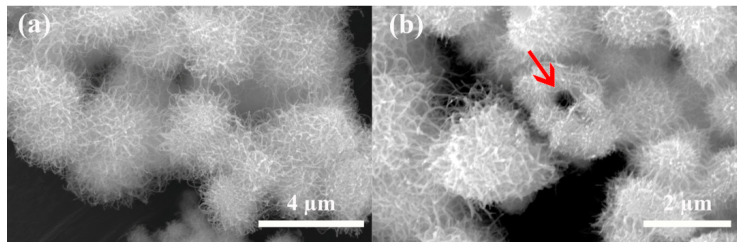
(**a**) SEM images of the as-prepared WO_3_; (**b**) SEM images of the as-prepared WO_3_ showing a hollow structure through the broken sphere (red arrow).

Scanning electron microscopy (SEM) images of the as-prepared pure WO_3_ are shown in Figure 2a. The as-prepared nanowires assembled into urchin-like hollow spheres with sizes ranging over 1.5–2 μm, and the hollow structure can be seen from the broken sphere clearly (Figure 2b). Instead of growing straight from the center as previously reported [18], the assembled nanowires grow around the shell by curling. To explore the synthesis process of urchin-like WO_3_ hollow spheres, several experiments were conducted to investigate the effects of hydrothermal time, hydrothermal temperature and additives on morphology. Detailed experimental procedures can be seen in supplementary documents (Figures S1–S3). In conclusion, a feasible mechanism was proposed. Firstly, WCl_6_ is hydrolyzed to W^6+^ in ethanol containing tiny droplets of ethylene glycol. Then, it will nucleate in ethanol and the interface between ethanol and ethylene glycol, resulting in a hollow structure. Such a formation process is also applicable to ZnO hollow microspheres [55]. After that, it will continue to grow into small pieces and then agglomerate into spheres at a suitable high temperature and high pressure, because of the principle of lowest energy. As the reaction continues, ethylene glycol could act as a capping agent to control the anisotropic growth of nanoflakes into nanowires under acidic condition. Thus, nanowires assembled urchin-like WO_3_ hollow sphere forms.

### 3.2. Structural Properties of the Prepared WO_3_

Figure S4 clearly shows thermograms of the prepared WO_3_. The thermogravimetric test was conducted to analyze the thermal stability of the products at elevated temperature. It is evident that three different stages of decomposition occur during the thermal analysis. In the first stage, 5.9% weight loss occurred because of the loss of common adsorbed water, which corresponds to the endothermic peak of 120 °C in DTA. The loss in the second stage (~3.3%) at around 400 °C is related to the evaporation of the bound water. After that, the weight of the sample decreased slightly. This is speculated to be related to the formation of oxygen vacancies due to the acceleration of molecular thermal movement at high temperature. Based on this, we annealed the samples at 400, 450, 500 and 550 °C for 2 h in air, respectively. The typical XRD pattern of the as-prepared and calcined materials are shown in Figure S5a; all XRD peaks can be well indexed to monoclinic I (γ-WO_3_) (JCPDS no. 83-0951), which is stable at room temperature [41]. Before annealing, the XRD pattern shows a broad peak, indicating the existence of some amorphous phases. After annealed, all diffraction peaks of γ-WO_3_ can be clearly observed. Based on the above results, γ-WO_3_ is stable here, and material properties will not change in future tests. As the annealing temperature increases, the line-widths of the Bragg lines of WO_3_ nanocrystals gradually become keen-edged, the crystallite sizes calculated by the Scherrer equation based on the (200) peak were 28, 38, 39 and 42 nm for W-400, W-450, W-500 and W-550, respectively [54]. 

**Figure 3:**
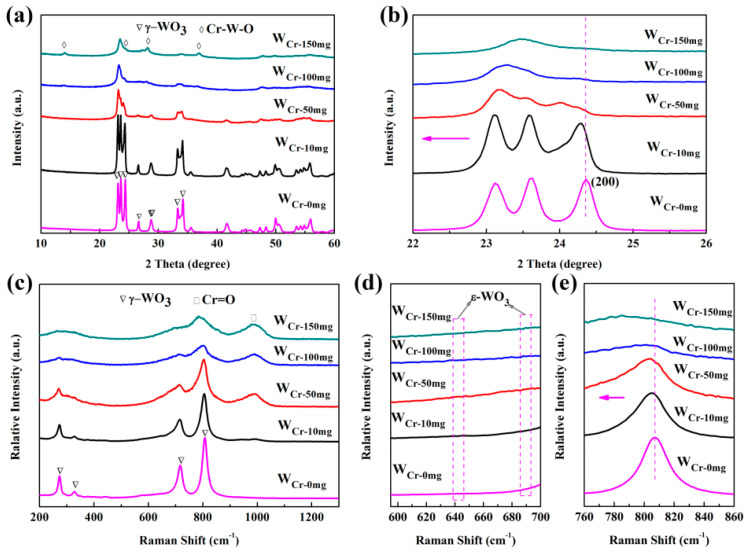
(**a**,**b**) X-ray powder diffraction (XRD) patterns, (**c**–**e**) Raman spectra of W_Cr-0mg_ - W_Cr-150mg_.

The XRD spectra of the Cr doped samples are shown in Figure 3a. Most of the diffraction peaks also correspond to monoclinic I (γ-WO_3_) and there are a couple of new peaks at 2θ = 13.8° and 36.5°, which correspond to chromium WO_3_ (JCPDS no. 43-0440). In addition, since Cr^3+^ (0.69 Å) and W^6+^ (0.62 Å) have similar ionic radii, it is reasonable to assume that Cr ions are incorporated into the WO_3_ lattice as a surrogate impurity. Furthermore, the intensities of diffraction peaks declined and the linewidths of diffraction peaks widened with an increasing amount of Cr, indicating that Cr dopants within WO_3_ caused degeneration of the crystallinity and reduction of crystallite size. When the Cr-doping amount is higher than 50 mg, the diffraction peaks widened and combined to form a broad hump. When closely observing Figure 3b, the observed shift in the (200) peak can be attributed to the small difference between the ionic radii of W^6+^ and Cr^3+^, and then leads to a slight distortion in the crystal lattice, which can produce a number of defects and change the gas response [56].

In order to confirm more structural information, Raman spectras of WO_3_ samples are investigated (seen in Figure S5b). The peaks at 272, 324, 715 and 806 cm^−1^ can be attributed to υ(O-W-O) or δ(O-W-O) stretching modes of γ-WO_3_, respectively [46]. Raman spectras for samples with different Cr-doping amounts are shown in Figure 3c, the band centered at 992 cm^−1^ can be assigned to the stretching mode of Cr = O terminal bonds of dehydrated monochromates, indicating the existence of chromium [57]. This peak becomes stronger as the amount of Cr-doping increases. According to previous literature [42], Cr doping will facilitate the formation of ε-WO_3_, which has strong interaction with the polar acetone molecule. By amplifying Figure 3c (seen in Figure 3d), there is no peak at 642 and 688 cm^−1^ which belong to ε-WO_3_. Additionally, compared with pure WO_3_, a mean blue shift is observed for the peak at 806 cm^−1^ (seen in Figure 3e), associated with shortening of O-W-O bonds, which corresponds to slightly smaller cell parameters of Cr-doped WO_3_ as compared to pure WO_3_ [56].

**Figure 4:**
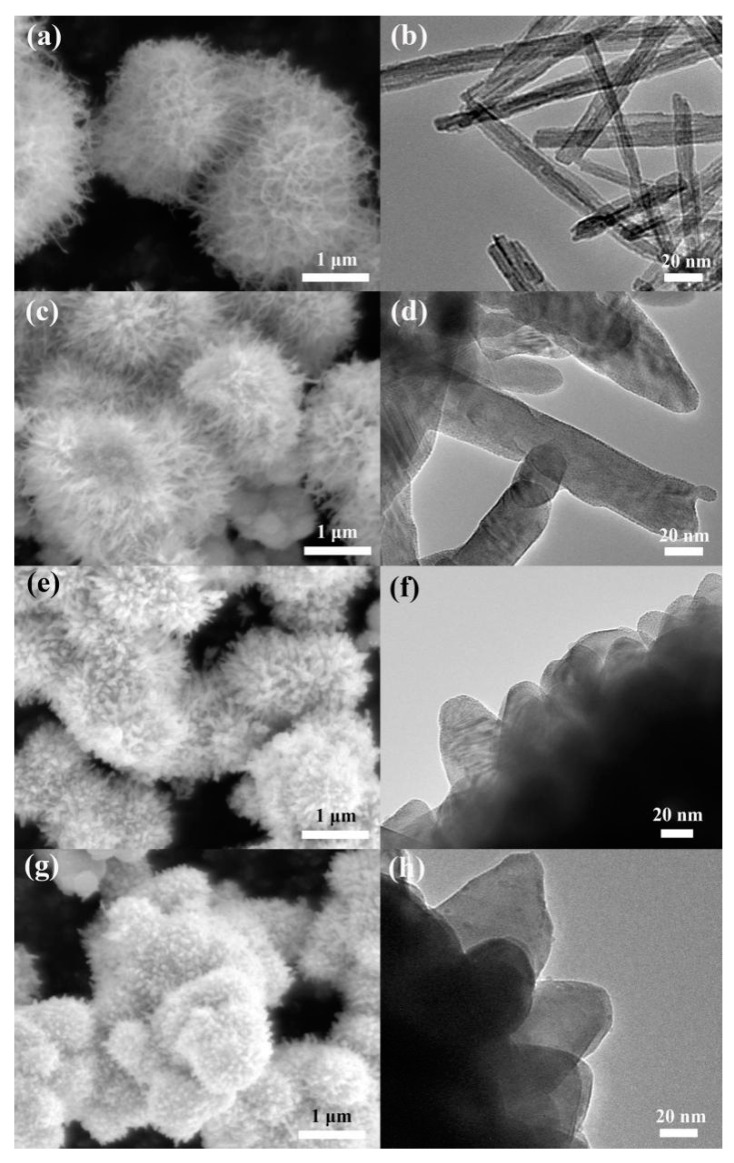
SEM (**a**,**c**,**e**,**g**) and TEM (**b**,**d**,**f**,**h**) images of W400, W450, W500 and W550.

Figure 4a–g presents SEM and transmission electron microscopy (TEM) images of annealed WO_3_ samples (W-400, W-450, W-500 and W-550). All the samples annealed below 450 °C exhibited similar morphologies. However, when the annealing temperature continues to rise, it is obvious that nanowires become shorter and thicker, and change to nanorods on the sphere’s surface, which greatly reduces the specific surface area of the material. It can be estimated that the diameters of W-400, W-450, W-500 and W-550 are 13, 30, 40 and 55 nm, respectively. Figure S6 confirms that the hollow structure still exists after annealing at 450 °C.

**Figure 5:**
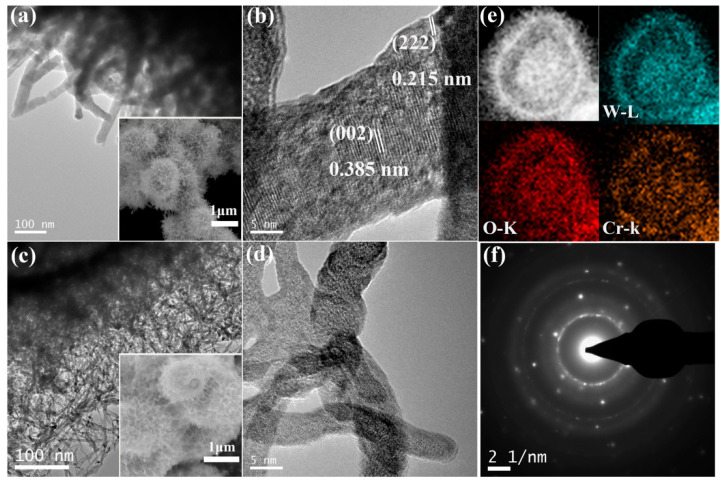
(**a**) TEM (**b**) high-resolution transmission electron microscopy (HR-TEM) images of W_Cr-0mg_; (**c**) TEM (**d**) HRTEM images of W_Cr-100mg_; (The inset images of a and c are the SEM images of W_Cr-0mg_ and W_Cr-100mg_, respectively.) (**e**) Elemental mapping for W, O, and Cr elements in W_Cr-100mg_; (**f**) selected area electron diffraction (SAED) pattern of W_Cr-100mg._

Figure S7 shows the SEM images of the Cr-doped WO_3_. The results show that the morphology of the material has not changed significantly under low concentration doping. When the doping amount reaches 100 mg, it grows like cotton outside the shell. However, the diameter of the assembled nanowires is shrinking as the doping amount increases. When the Cr doping amount reaches 150 mg, the diameter of the nanowires are so small that they fuse into small pieces and tightly pack into a sphere, indicating a decrease in specific surface area. Comparing samples annealed at 450 °C before and after doping, as seen in Figure 5, the diameter of the assembled nanowires changes from 30 to 5 nm. The lattice fringes of W-450 with d-spacing of 0.385 and 0.215 nm correspond to the (002) and (222) planes of γ-WO_3_, while the crystallinity of Cr-doped WO_3_ became worse, which is consistent with the conclusion of XRD and Raman. The elemental mapping shown in Figure 5e proves the uniform distribution of W, O, and Cr elements in W_Cr-100mg_; Figure 5f shows the corresponding selected area electron diffraction (SAED) pattern of W_Cr-100mg_ which ascertains that the sample is polycrystalline structure in nature.

Generally, in the urchin-like spheres, thinner nanowires indicate a higher number density. Such a structure will provide a higher surface area for a gas sensing reaction. Related models have been discussed before [18]; the specific surface area of an urchin-like microsphere is expressed as follows:

(1) $$S=\frac{n\left(2\pi \mathrm{rh}+{\pi r}^{2}\right)+4{\pi R}^{2}\left(1-\theta \right)}{n\left({\pi r}^{2}h\right)\rho +\left(4/3\right){\pi R}^{3}\rho }$$

where r, h, θ and ρ are the nanowires’ diameter (d = 2r), length, coverage and density, respectively. R indicates the spherical nucleus’ radius. The result implies that the surface area of urchin-like microspheres is inversely proportional to the nanowires’ diameter (d). Therefore, the surface area of the Cr-doped WO_3_ is increased compared to the pure one.

**Figure 6:**
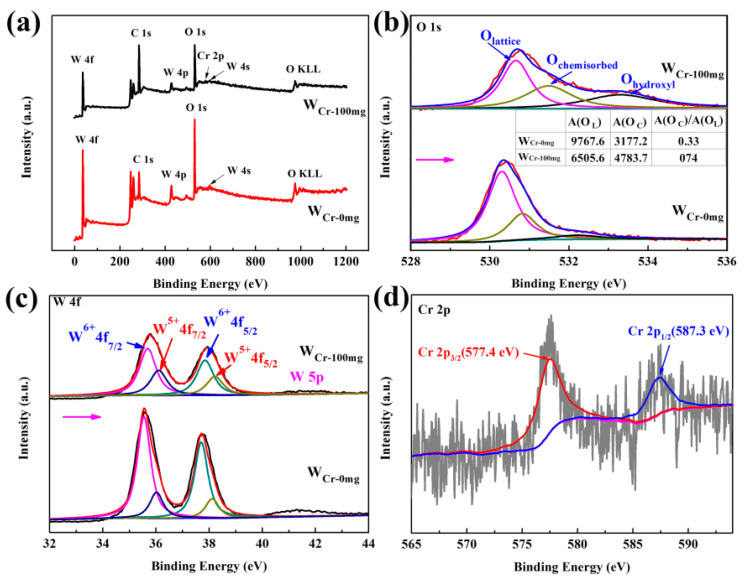
X-ray photoelectron spectroscopy (XPS) spectra of W_Cr-0mg_ and W_Cr-100mg_: (**a**) full survey spectrum, (**b**) O 1s, (The inset table displays the values of the respective peak areas of O_lattice_ and O_chemisorbed_ and the proportion of O_chemisorbed/_O_lattice_ in pure and Cr-doped WO_3_). (**c**) W 4f, (**d**) Cr 2p.

XPS analysis was carried out to further investigate the surface compositions and oxidation states of the pure and Cr-doped WO_3_ spheres. The comparison of their full-range XPS spectrum (Figure 6a) reveals the presence of Cr. The high-resolution XPS spectra of O1s peaks in Figure 6b could be deconvoluted into three major peaks. The main peak at 530.3 eV can be ascribed to the lattice oxygen (O_lattice_). The minor peak at 530.8 eV can be assigned to the chemisorbed oxygen (O_chemisorbed_) on the WO_3_ surface, which has a large influence on gas sensitivity [39]. The peak at 532.2 eV is ascribed to hydroxyl surface groups (O_hydroxyl_) due to water adsorption [39]. As seen in the inset images of Figure 6b and Figure S8a, the proportion of O_chemisorbed_/O_lattice_ is calculated to be 0.33 for pure WO_3_ and 0.74 for Cr-doped WO_3_, and it goes from 0.26 to 0.68 as the annealing temperature rises, indicating an increase in oxygen vacancies. In addition, O_hydroxyl_ also increases due to the defects caused by annealing or doping and thus facilitates the adsorption of water molecules. The high-resolution spectra of W 4f (Figure 6c, Figure S8b) can be deconvoluted into two doublet pairs at binding energies of 35.5–37.7 and 36.0–38.1 eV, which correspond to W 4f_5/2_-W 4f_7/2_ of W^6+^ and W^5+^ [46], respectively. The results show that increasing annealing temperature and doping amounts will lead to the increase of W^5+^, corresponding to an increase of oxygen vacancy and chemisorbed oxygen, which is consistent with the high-resolution XPS spectra of O1s. The Cr 2p XPS spectrum (Figure 6d) contains doublet pairs with binding energies of 577.36 and 587.26 eV, which are assigned to Cr 2p_3/2_ and Cr 2p_1/2_, respectively [58].

### 3.3. Gas Sensing Properties

**Figure 7:**
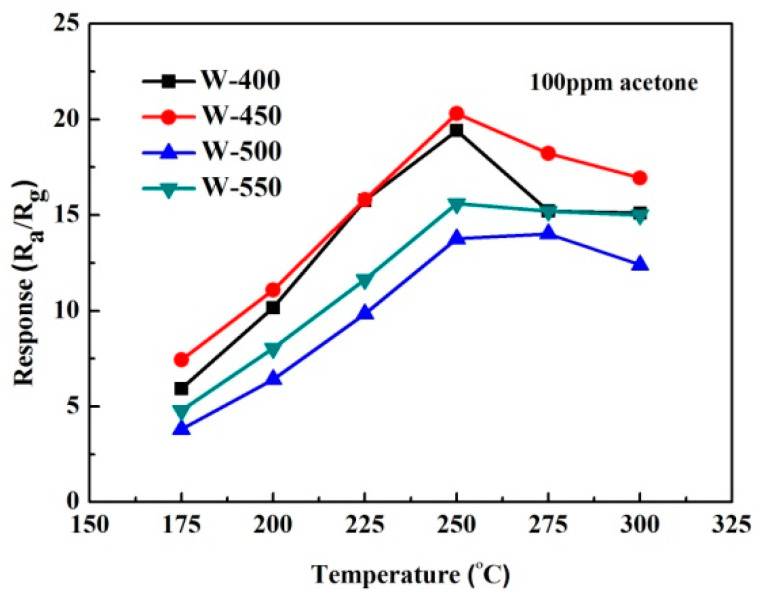
The responses of W-400, W-450, W-500 and W-550 gas sensors as a function of the operating temperature to 100 ppm acetone.

In order to explore the influence of the contact interface between sensing materials and electrodes on gas performance, the I-V curve of W-450 sensors was recorded while varying the voltage from −0.1 to 0.1 V, showing a linear response (see Figure S9 in the Supplementary Materials). Therefore, the influence of the electrical contacts on the observed sensor response can be excluded. To investigate the effect of annealing temperature on gas sensitivity, the sensors based on the samples (W-400, W-450, W-500 and W-550) were tested toward 100 ppm acetone at different temperatures ranging from 175 to 300 °C (seen in Figure 7). The response of the samples increased with working temperature up to around 250 °C, and then decreased. Previous works have proved that the reaction between acetone and the surface of γ-WO_3_ cannot be realized at room temperature, unless there is some external energy supply, such as higher temperature [46]. The surface reaction rate and gas adsorption rate accelerate as the operating temperature increases. When the device works at a certain critical temperature, the response reaches the maximum, because of the balance of adsorption-desorption rate and well-suited activation energy. When the operating temperature is too high, the desorption rate of the gas molecules is too high to react before escaping the active centers of surface, which results in a decreased response [59]. The sensor coated with W-450 exhibits the largest response (20.32) to 100 ppm acetone at 250 °C, and the response of W-400 (19.42) is a little bit lower than W-450, while the response of W-500 (13.77) and W-550 (15.60) sensors are significantly decreased. Since the material annealed at 450 °C performs better than the other ones, we should suppose that other doped materials are also annealed at 450 °C.

**Figure 8:**
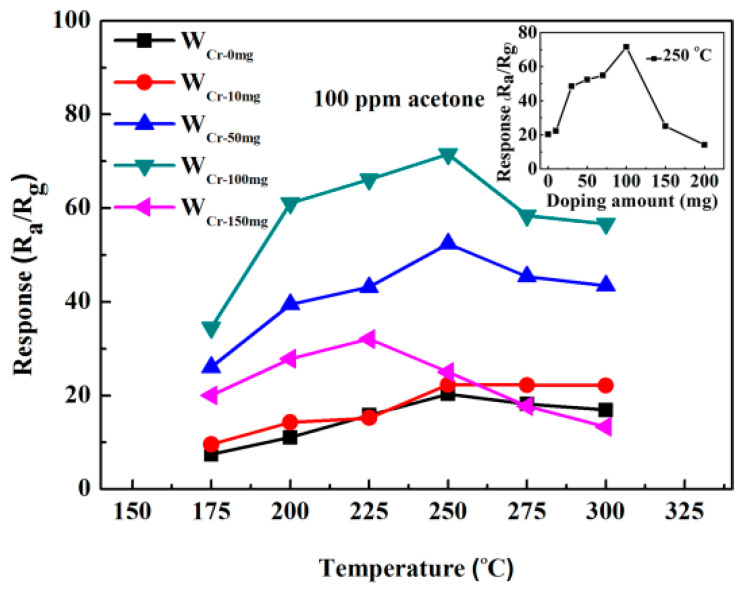
Responses of W_Cr-0mg_, W_Cr-10mg_, W_Cr-50mg_, W_Cr-100mg_ and W_Cr-150mg_ gas sensors as a function of the operating temperature, to 100 ppm acetone. (The inset image is the response of different Cr doping amount gas sensors to 100 ppm ethanol at 250 °C).

The effect of the Cr doping amount on sensing properties was further investigated. Figure 8 demonstrates the responses of W_Cr-0mg_, W_Cr-10mg_, W_Cr-50mg_, W_Cr-100mg_ and W_Cr-150mg_ sensors to 100 ppm acetone as a function of operating temperature. With the increase of working temperature and Cr doping amount, the responses increase firstly and then decrease. The optimum operating temperature for all doped samples is 250 °C except for W_Cr-150mg_. For W_Cr-150mg_, its optimal operating temperature is reduced to 225 °C, which indicates that chromium doping can reduce the activation energy of the reaction between the acetone molecule and the material’s surface. At the optimum operating temperature, W_Cr-100mg_ presents the highest response of 71.52 while W_Cr-0mg_, W_Cr-10mg_, W_Cr-50mg_, and W_Cr-150mg_ have the responses of 20.32, 22.33, 52.42 and 32.08, respectively. The base resistance of the materials increases with increasing doping amount at the same temperature. As the working temperature increases, the base resistance will decrease accordingly (seen in Figure S10). When the doping amount reaches 150 mg, the crystallinity and carrier mobility of the material become very poor, which is unfavorable to the transport of carriers during the sensing process and leads to response reduction.

**Figure 9:**
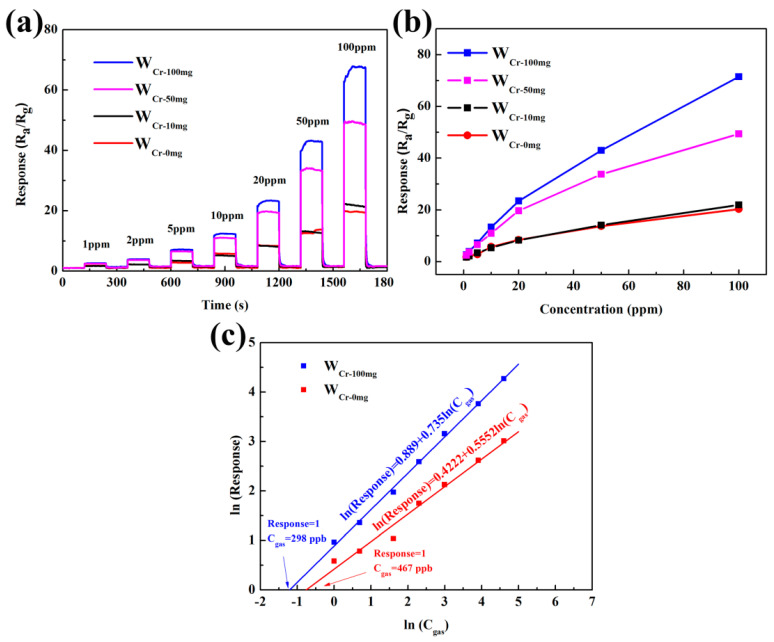
(**a**) Sensing transient curve of the sensors based on W_Cr-0mg_, W_Cr-10mg_, W_Cr-50mg_ and W_Cr-100mg_ to different concentrations of acetone ranging from 1 to 100 ppm measured at 250 °C at the relative humidity (RH) of 40%. (**b**) The corresponding responses to different acetone concentrations for the sensors. (**c**) The relationship between ln(Response) and ln(C_gas_).

The dynamic response curves of all sensors to acetone concentration from 1 to 100 ppm at 250 °C are shown in Figure 9a. W_Cr-100mg_ has the response of 2.62 to 1 ppm acetone and 3.88 to 2 ppm acetone, while the responses of W_Cr-0mg_ are 1.79 and 2.19. Diabetes detection requires that the difference in response to exhaled gas between the healthy people (acetone concentration: below 1 ppm) and patients (about 2 ppm) should be as large as possible. Thus, the increment of 1.26 may allow reliable diagnosis of diabetes for W_Cr-100mg_, compared with the 0.40 for W_Cr-0mg_. As shown in Figure 9b, it is obvious that the responses of sensors exhibit a positive correlation property with gas concentrations, and it is almost linear at relatively low concentrations (1–10 ppm). The experimental data can be fitted by the typical power relation for metal oxide sensors [60]:

Response = k(C_gas_)^β^, (2)

where k, C_gas_ and β is a constant, the gas concentration and power-law exponent, respectively. The detection limit is calculated taking 1 as the minimum response to produce an appreciable signal in the detection of a specific gas. The results show that acetone detection limit is identified as 298 and 467 ppb, respectively (seen in Figure 9c).

**Figure 10:**
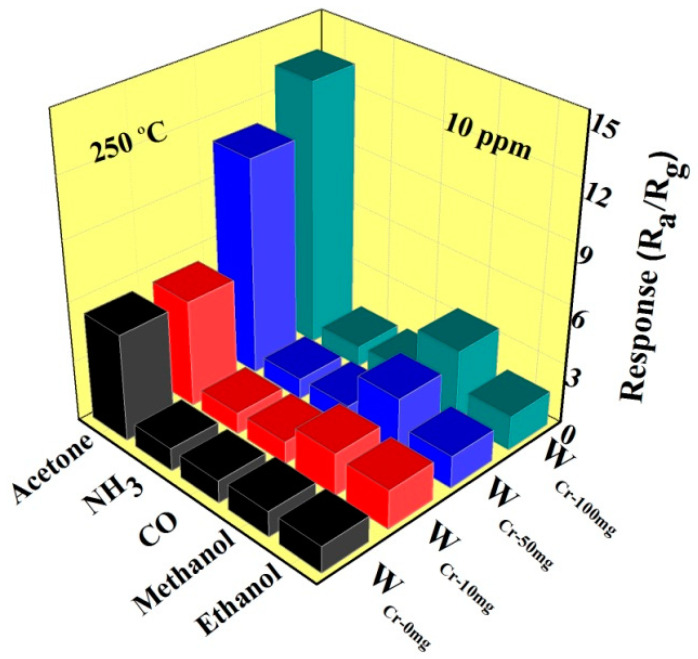
The response of W_Cr-0mg_, W_Cr-10mg_, W_Cr-50mg_ and W_Cr-100mg_ gas sensors to 10 ppm of various target gases at 250 °C.

There are other typical biomarkers in the exhaled gas such as CO, NH_3_, ethanol and methane [4,5,6,7,8]. In order to exclude the effects of these gases, the sensor was tested toward 10 ppm of different gases at 250 °C. It can be seen from the Figure 10, the response to acetone is quite excellent for the undoped WO_3_ hollow urchin-like spheres, as compared to the other gases. This suggests that γ-WO_3_ is more beneficial for the adsorption of acetone, because of the smaller equilibrium distance and the higher adsorption energy, thus a stronger charge transfer between γ-WO_3_ and acetone molecular [61]. When Cr element is doped, the response to ethanol and methanol is increased to some extent, and there is almost no response to CO and NH_3_. It is worth noting that the response of W_Cr-100mg_ to 100ppm acetone is 3.5 times greater than that without doping, but its response to other gases has not improved.

**Figure 11:**
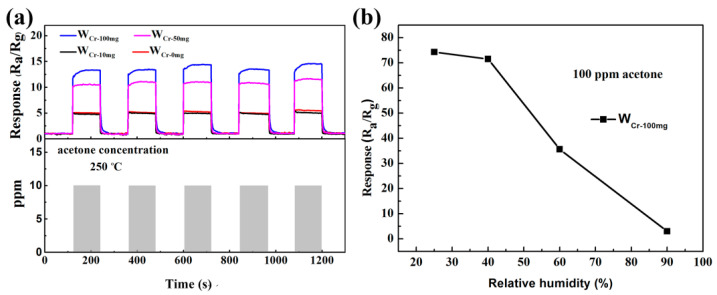
(**a**) Five cycles of response of W_Cr-0mg_, W_Cr-10mg_, W_Cr-50mg_ and W_Cr-100mg_ gas sensors towards 10 ppm acetone at 250 °C, and (**b**) the corresponding responses to different relative humidities (%) for W_Cr-100mg_ sensors.

The repeatability of the sensor is the key factor whether the sensor can be applied in practical life. As shown in Figure 11a, the sensors were exposed to 10 ppm of acetone for 5 times at optimal operating temperature (250 °C). The result shows that a corking repeatability was obtained, with less than 2% response variation. Moreover, human exhaled breath contains large amounts of N_2_, CO_2_, O_2_, and water. The sensitivity of metal oxide sensors will decrease due to the absorption and dissociation of water molecules on the oxide surface, and the following reactions will occur: [62]

2H_2_O + O_o_→3OH^−^ + H^+^. (3)

After this reaction, an ionic conductive layer will be formed on the surface of the metal oxide, leading to improved conductivity, which is supported by experimental results (Figure S11). In addition, water molecules adsorbed on the material’s surface will compete with gas molecules to occupy the active sites, and prevent the electron transfer between the gas molecules and the material when the concentration of water molecules is too high. The response will decrease with increasing humidity. Figure 11b displays W_Cr-100mg_’s response curve with the change of humidity to 100 ppm acetone. It shows that the response decreases to 35.65 when the humidity reaches 60%, which is still higher than the undoped WO_3_. When the RH is 90%, the response is only about 3. This problem is usually solved by using a water trap or water-adsorbent materials placed before the sensing surface, to reduce the amount of water and therefore to enhance the response to acetone [63]. In brief, sensors made from W_Cr-100mg_ have a good sensitivity, low response to other gases and good repeatability for the detection of acetone. 

The acetone sensing performance of the WO_3_-based sensors reported previously and in this work is shown in Table 1. Obviously, the optimal operating temperature of sensors fabricated in this work is lower than other sensors. Furthermore, the gas response of the W_Cr-100mg_ sensor is also relatively high in these sensors. In conclusion, the sensors based on Cr-doped urchin-like WO_3_ hollow spheres have an excellent advantage for acetone detection.

### 3.4. Sensing Mechanism

**Figure 12:**
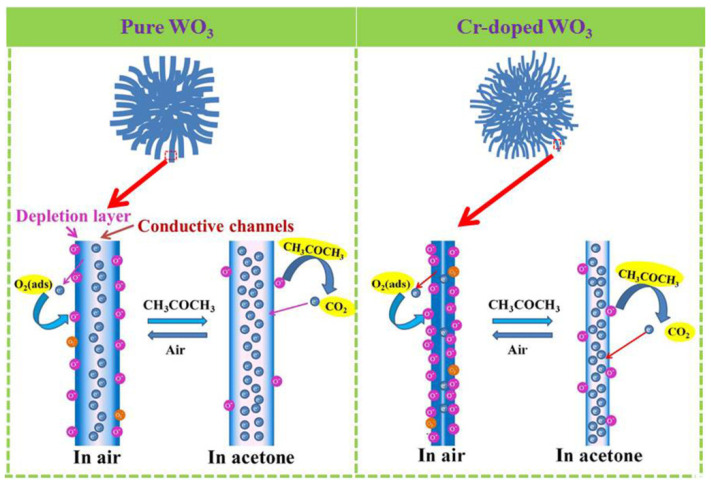
Representative models for acetone sensing mechanisms of: (**a**) pure and (**b**) Cr-doped WO_3_ at 250 °C.

The resistance-type gas sensor monitors resistance variation caused by adsorbed oxygen and gas molecular. In this mechanism, oxygen molecular in the air is chemically adsorbed on the surface of oxides and then captures electrons to form chemisorbed O_2_^−^, O^−^, and O^2−^ ions, which depend on working temperature, causing depletion region layers on their surfaces. When oxidizing gas like NO_2_ or reducing gases like acetone are introduced, the target gas molecules will react with the surface of the material and extract/release more electrons from/to the conduction band, leading to an increase/decrease in resistance. In our work, the mechanism of WO_3_ is represented in Figure 12. When the sensor is exposed in air, the adsorption process could be described as follows [65]:

O_2_ (gas)→O_2_ (ads), (4)

O_2_ (ads) + e^−^ →O_2_^−^ (ads) (T_op_ <100 °C), (5)

O_2_^−^ (ads) + e^−^ →2O^−^ (ads) (100 < T_op_ < 300 °C), (6)

O^−^ (ads) + e^−^ →O^2−^ (ads) (T_op_ > 300 °C). (7)

Upon exposure to acetone, the reaction could not occur at room temperature because of its high activation energy. As the operating temperature is elevated to 250 °C, acetone begins to react with pre-adsorbed oxygen ions (O^−^) or bulk oxygen as follows [44]:

CH_3_COCH_3_ (gas) + O^−^ (ads) → CH_3_COC^+^H_2_ + OH^−^ + e^−^ (8)

CH_3_COCH_3_ (gas) + O (bulk) + OH^−^ → CH_3_COOH +V_O_ + CH_3_O (9)

or

CH_3_COCH_3_ (gas) + O^−^(ads) → CH_3_C^+^O + CH_3_O^−^ + e^−^ (10)

CH_3_C^+^O → C^+^H_3_ + CO (11)

CO + O^−^ (ads) → CO_2_ + e^−^ (12)

Therefore, electrons are released back to the conduction band, causing an increase in the resistance of the material, that is, a response.

According to the above mechanism, carrier transfer is the key to the reaction, which depends on the equilibrium distance and the adsorption energy between the gas and material surface [61]. Previous studies have proved that γ-WO_3_ has a higher response to acetone than other gases, which indirectly proved that it has a smaller equilibrium distance and a higher adsorption energy, thus, it illustrates a stronger charge transfer between γ-WO_3_ and acetone molecular. Moreover, the response also largely depends on the proportion of the depletion layer width in the overall material, and it is related to the specific surface area and porosity. 1–3 urchin-like γ-WO_3_ hollow spheres possess a high surface area, and they can provide plenty of adsorption sites for acetone molecular, resulting in enhanced gas response [66]. With the increasing of annealing temperature, the diameter of nanowires significantly increased and the shell became more compact, as seen from SEM and TEM images, leading to the reduction of the specific surface area and gas diffusion velocity and thus weakened response. When Cr-100 mg is doped, the diameter of the assembled nanowires decreases from 30 to 5 nm, leaving the entire nanowire in a depleted state. This is one of main reasons for the enhanced response.

Another factor that affects the width of the depletion layer is the content of oxygen vacancy in the sample [58]. The formation of oxygen vacancy needs to overcome a certain amount of energy. For WO_3_, high temperature annealing will cause oxygen separation in the lattice, resulting in oxygen loss and the formation of oxygen vacancy. Cr doping can also lead to oxygen vacancy formation, the equation of defect reaction after adding Cr^3+^ is as follows:

(13) $${\mathrm{Cr}}_{2}O_{3}\overset{{\mathrm{WO}}_{3}}{\to }2{\mathrm{Cr}}_{w}\prime \prime \prime +3O_{O}+3V_{O}{}^{¨},$$

where we have adopted the Kroger-Vink’s notation for the defects:${\;\mathrm{Cr}}_{w}{}^{\prime \prime \prime }$ is Cr substitution in W sites with three negative charges, ${\;V}_{O}{}^{¨}$ represents oxygen vacancies with two positive charges. The positively charged oxygen vacancies will capture electrons from the conduction band through electrostatic interaction and act as the adsorption center for chemisorbed oxygen ions on the surface, so that more electrons can be captured from the conduction band. When reducing gas is introduced, even a small amount of electrons release can also cause a relatively high resistance change. The combination of these two factors makes the material have an optimal annealing temperature and suitable Cr doping concentration.

The reason for the favorable effect of Cr dopants for acetone detection can be ascribed to the stabilized ε-phase, which can greatly improve the acetone response [43], since ε-phase WO_3_ does not form. Compared with previous literature [42,45], Cr-doped WO_3_ is synthesized by hydrothermal in this work, while the Cr-doped WO_3_ containing ε-phase is prepared by RF thermal plasma or flame spray pyrolysis. Therefore, in addition to chromium doping, the rapid temperature change might be a key to the existence of ε-phase. In this case, the enhancement effect of Cr dopants can be explained from a new perspective. The UV-visible absorption spectra of W_Cr-0mg_ and W_Cr-100mg_ were measured, and converted into a Tauc plot to determine the optical band gap by the following equation:

(αhν)^2^ = A(hν − E_g_). (14)

**Figure 13:**
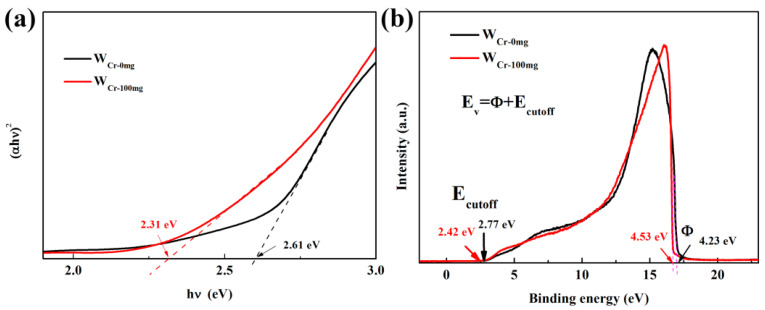
(**a**) (αhν)^2^-hν curves of pure and Cr-doped WO_3_, and (**b**) the valence band of pure and Cr-doped WO_3_ measured by ultraviolet photoelectron spectroscopy (UPS) (He I excitation, hν = 21.22 eV) spectra.

As shown in Figure 13a, the extrapolation of the hν-(αhν)^2^ plot on x intercepts gives the optical band gaps (E_g_) of 2.61 and 2.31 eV for W_Cr-0mg_ and W_Cr-100mg_, respectively. The decrease in optical band gap can be attributed to the change of electronic structure and the introduction of impurity level in the forbidden band after the addition of Cr. It means that more electrons are excited from the valence band and impurity level into the conduction band at relatively high temperatures, and then trapped by adsorbed oxygen, leading to an increase in depletion layers. This increase causes a reduction of conductive channels and an increase in basal resistance. The values of the valence band (E_v_) and the conduction band (E_c_) are determined by the following equation on the basis of UPS (Figure 13b):

E_v_ = Φ + E_cutoff_ (15)

E_c_ = E_v_ − E_g._ (16)

**Figure 14:**
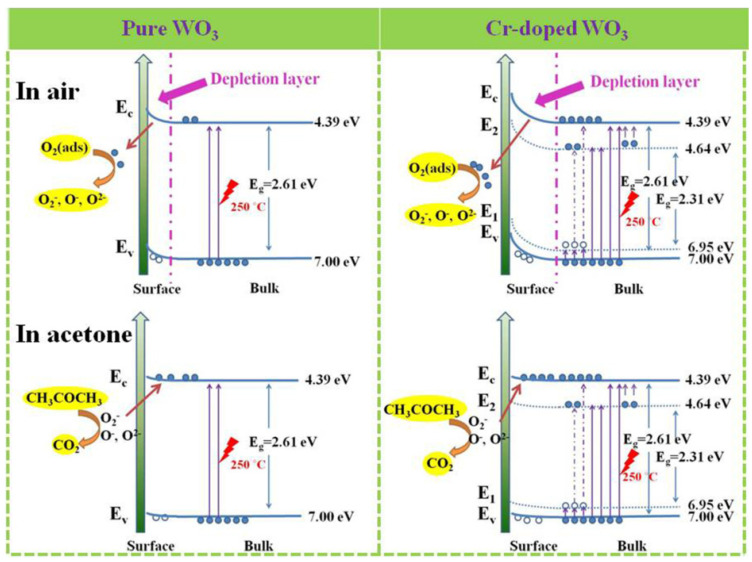
The schematic band diagram of: (**a**) pure WO_3_ and (**b**) Cr-doped WO_3_ before (up) and after (down) detecting acetone vapor.

The work function (Φ) was calculated by using He I excitation energy (hν = 21.22 eV) minus the big intercept of the curve on the abscissa axis. The derived E_v_ of the pure and Cr-doped WO_3_ are 7.00 and 6.95 eV, respectively, and the corresponding E_c_ come out to be 4.39 and 4.64 eV. Based on the above data, the schematic diagram of response mechanism is shown in the Figure 14. Cr doping not only introduces the acceptor energy level (E_1_) near the valence band, but also introduces the impurity energy level (E_2_) near the conduction band, which can intercept heat excited electrons in the conduction band and cause an increase in basal resistance as a trap center. The introduction of E_1_ can be attributed to the formation of oxygen vacancy caused by doping, which proves that in addition to the oxygen vacancy generated by annealing, doping will also produce more oxygen vacancy in the lattice. The introduction of E_2_ means that when the working temperature increases, the trapped electrons in the trap center will be thermally excited to the conduction band and captured by adsorbed oxygen. In other words, it can reduce the recombination rate of electron-hole pairs, so that more electrons can be captured by adsorbed oxygen. Finally, considering the lower working temperature of W_Cr-150mg_, it means that the energy required for surface reaction is reduced, which can be attributed to the catalytic effect of Cr doping.

## 4. Conclusions

In this work, 1–3 urchin-like WO_3_ hollow spheres with diameters ranging from 1.5 to 2 μm were successfully synthesized via a simple hydrothermal method and followed by a series of annealing operations. Results show that pure WO_3_ annealed at 450 °C had the best response (20.32) to 100 ppm acetone at optimum operating temperature (250 °C), due to the relatively larger specific surface area and more oxygen vacancy. After doping with Cr, the sensor’s response to acetone was three times higher than that of the pure WO_3_ sensor, and its response to other gases was relatively low. Furthermore, it also exhibits lower detection limits (ppb-level) and excellent repeatability. The sensitivity of Cr-doped WO_3_ greatly improved because Cr ions can modulate the crystal growth and reduce the diameter of the assembled nanowires. Doping of Cr ions into WO_3_ nanocrystals can regulate the energy band structure to some extent by introducing an impurity energy level with a trap effect, and act as a catalyst to reduce the activation energy of the reaction. The improved sensor performance is also attributed to increased oxygen vacancy in the lattice. The excellent performance indicates that it could be a promising material for acetone detection in noninvasive diagnosis of diabetes.

## Figures and Tables

**Table 1:** Comparison of gas-sensing properties of sensors based on various sensing materials toward acetone.

Materials	Crystalline Phase	T_op_ (°C)	Acetone (ppm)	Response (S)	Ref.
Cr-doped WO_3_ particles	monoclinic γ- and ε-WO_3_	400	1	4.0	[42]
Si-doped WO_3_ nanoparticles	monoclinic γ- and ε-WO_3_	400	0.6	4.6	[44]
C-doped WO_3_ hollow spheres	monoclinic γ- and ε-WO_3_	300	10	11.0	[46]
Co-doped WO_3_ flower-like nanostructures	monoclinic γ-WO_3_	350	100	103.0	[47]
g-C_3_N_4_ modified WO_3_ nanosheets	monoclinic γ- and ε-WO_3_	340	100	35.0	[60]
Pt-WO_3_ Hemitube Networks	monoclinic γ-WO_3_	300	2	4.11	[64]
Cr-doped urchin-like WO_3_ hollow spheres	monoclinic γ-WO_3_	250	10	13.3	This work

## Supplementary Materials

The following are available online at https://www.mdpi.com/1424-8220/20/12/3473/s1, Figures S1–S3: The effects of hydrothermal time, hydrothermal temperature and additives on morphology, Figure S4: TGA image of the products, Figure S5: XRD and Raman spectra of samples annealed at different temperatures, Figure S6: SEM images of the hollow structure of the samples after annealing, Figure S7: the morphology doped with different amounts of Cr, Figure S8: XPS of samples annealed at different temperatures, Figure S9: I-V curve of sample, Figures S10–S11: base resistance of samples at different temperatures and humidities.
