# Supplementary material for: Cr-Doped Urchin-Like WO3 Hollow Spheres: The Cooperative Modulation of Crystal Growth and Energy-Band Structure for High-Sensitive Acetone Detection
Source: Sensors (Basel). 2020 Jun 19;20(12):3473. doi: 10.3390/s20123473 (PMC7348964; doi:10.3390/s20123473)
Supplement: Supplementary file 1 [file sensors-20-03473-s001.pdf]

sensors

Article

Cr-Doped Urchin-Like WO_3 Hollow Spheres: The Cooperative Modulation of Crystal Growth and Energy-Band Structure for High-Sensitive Acetone Detection

Qiongling Ding, Yanrong Wang *, Pengqian Guo, Jianjun Li, Chen Chen, Ting Wang, Kai Sun and Deyan He

[1] School of Physical Science and Technology, Lanzhou University, Lanzhou 730000, China; dingql18@lzu.edu.cn (Q.D.); guopq15@lzu.edu.cn (P.G.); jjli19@lzu.edu.cn (J.L.); chench2018@lzu.edu.cn (C.C.); wangting2017@lzu.edu.cn (T.W.); sunk2019@lzu.edu.cn (K.S.); hedy@lzu.edu.cn (D.H.)

* Correspondence: wyr@lzu.edu.cn

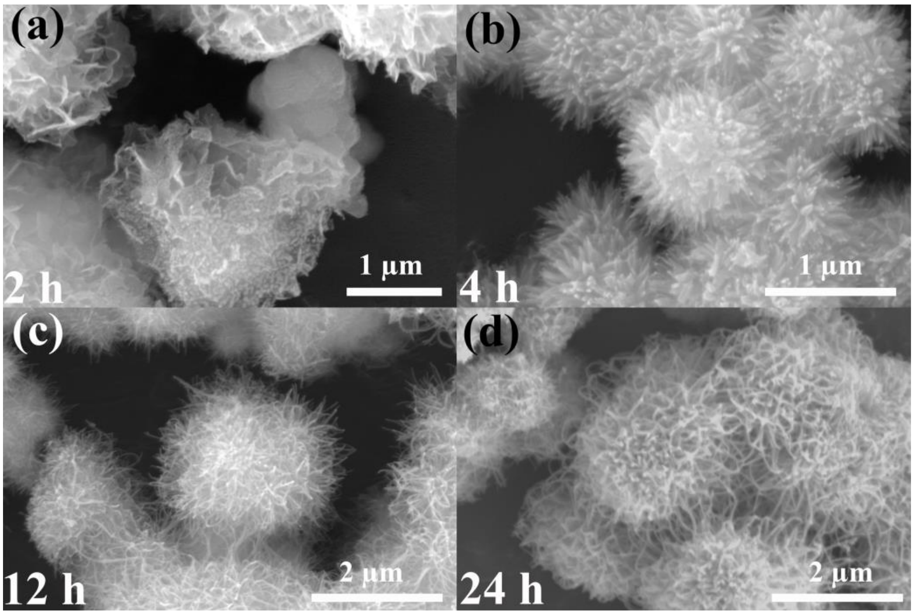

Figure S1. SEM images of products grown for (**a**) 2 h, (**b**) 4 h, (**c**) 12 h, (**d**) 24 h, respectively.

Time-dependent experiments found that the morphology will change with the increase of hydrothermal time (as shown in **Figure S1**). At the beginning of the reaction (2 h), ultra-thin crumpled nanoflakes were assembled as flower-like spheres, with diameters of about 1μm. When the reaction time increased to 4 h, urchin-like hollow spheres with outward-facing nanorods formed because of anisotropy consumption and growth of the nanoflakes. As the reaction time continually increased, nanorods grew to a long curly nanowire and the urchin-like spheres got bigger and puffier gradually; when the reaction time reached 24 h, the diameter of the hollow sphere increased to 2 μm. All of the above experiments were conducted at 180 °C.

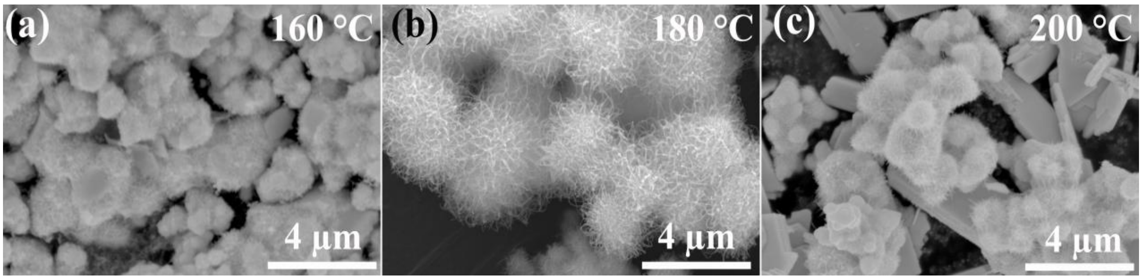

Figure S2. SEM images of products grown for 12 h at different hydrothermal temperatures: (**a**) 160 °C, (**b**) 180 °C, (**c**) 200 °C.

Temperature-dependent experiments found that, if the hydrothermal temperature was changed, the morphology would change accordingly (as shown in **Figure S2**). The morphology of as-obtained spheres conducted at 160 °C was very irregular; when the temperature increased to 200 °C, the urchin-like spheres coexisted with micron sheets.

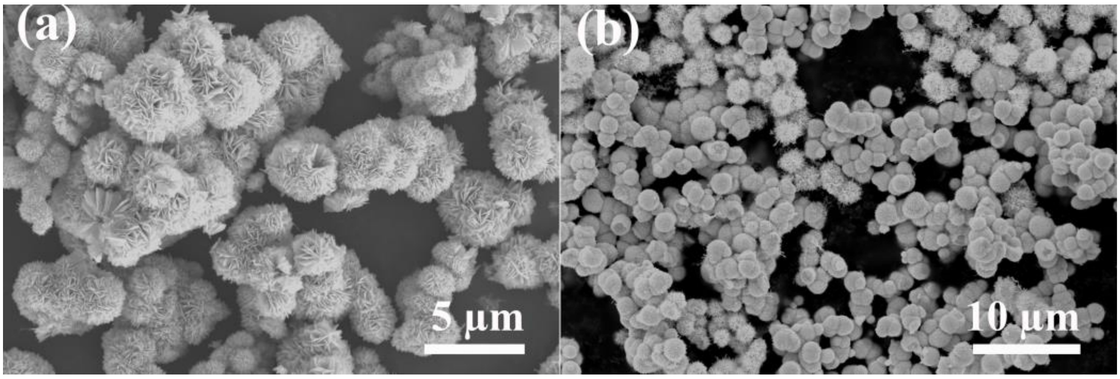

Figure S3. SEM images of products grown for 12 h at 180 °C without adding: (**a**) ethylene glycol, (**b**) HCl.

The role of additives in hydrothermal systems is particularly important; it can change the acidity, alkalinity and solubility of the precursor, and then change the crystallization and solubility of solute molecules. In addition, it can act as a capping agent which could preferentially adsorb on the specific crystal to vary the assembled mode of solute molecules. Keeping the hydrothermal time (12 h) and hydrothermal temperature (180 °C) unchanged, urchin-like spheres will became nanoflowers composed of nanosheets if no ethylene glycol was added in the precursor solution (seen in **Figure S3a**). In addition, no hollow structures were found, suggesting that ethylene glycol is the key to the formation of hollow structures. Ethylene glycol would form many tiny droplets in ethanol, and the nucleation occurred primarily at the interface between ethanol and ethylene glycol, resulting in a hollow structure. In the absence of hydrochloric acid, nanoparticles assembled nanospheres exist with small amounts of urchin-like spheres (**Figure S3b**). This indicated the importance of pH for oriented growth of crystals.

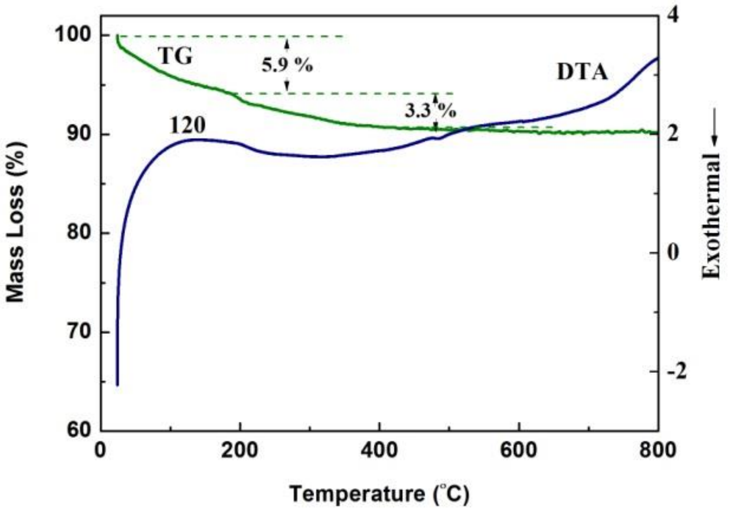

Figure S4. Thermal gravimetric analysis (TGA) image of the products in air by heating up from room temperature to 800 ℃.

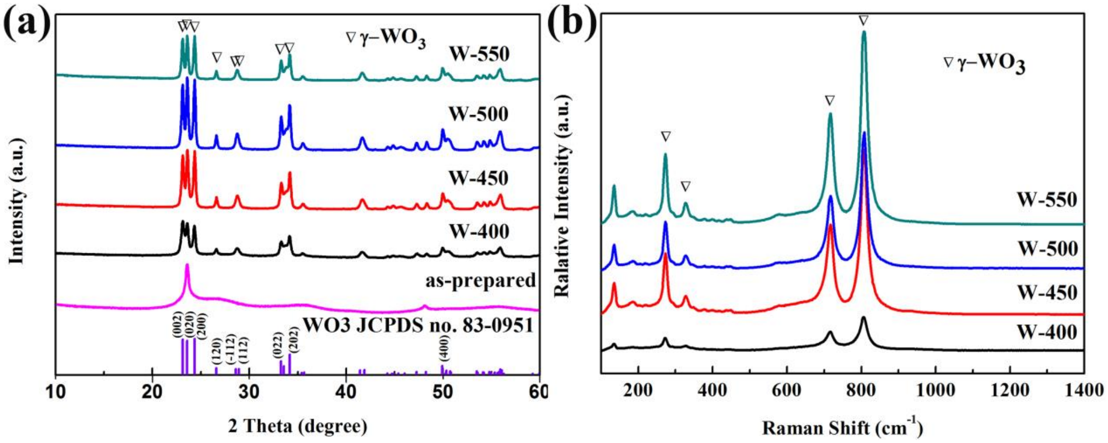

Figure S5. (**a**) XRD pattern (**b**) Raman spectra of W-400, W-450, W-500 and W-550.

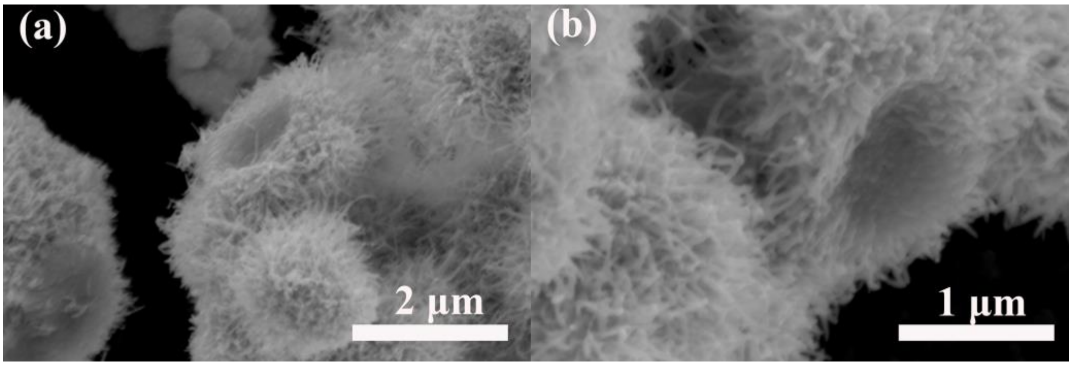

Figure S6. SEM images of the hollow structure of the samples after annealing.

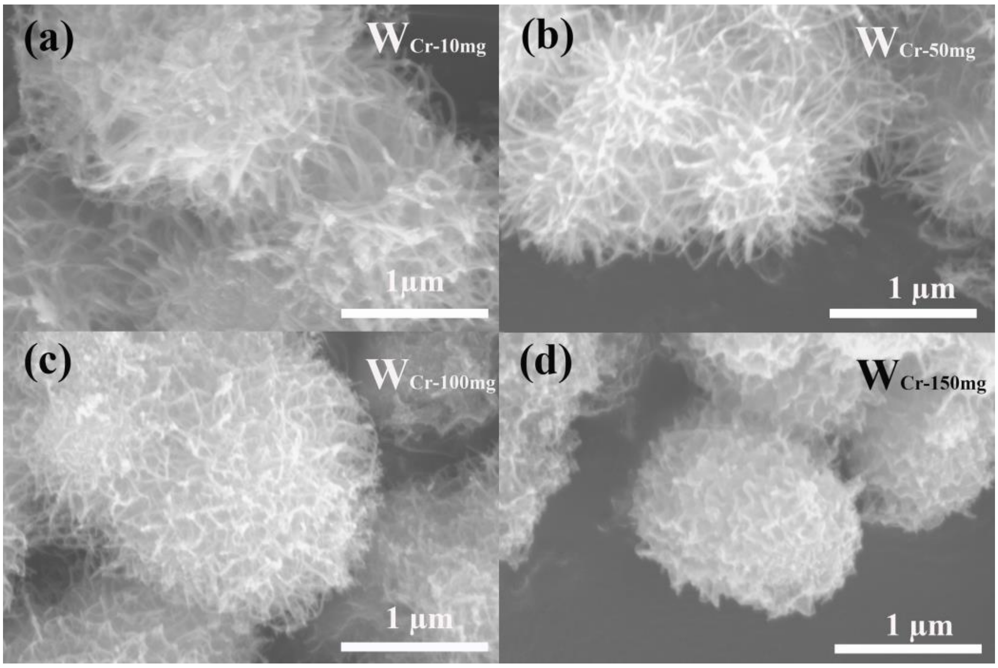

Figure S7. SEM images of (**a**) W_{Cr-0mg}, (**b**) $W_{Cr-10mg}$, (**c**) $W_{Cr-50mg}$, (**d**) $W_{Cr-100mg}$.

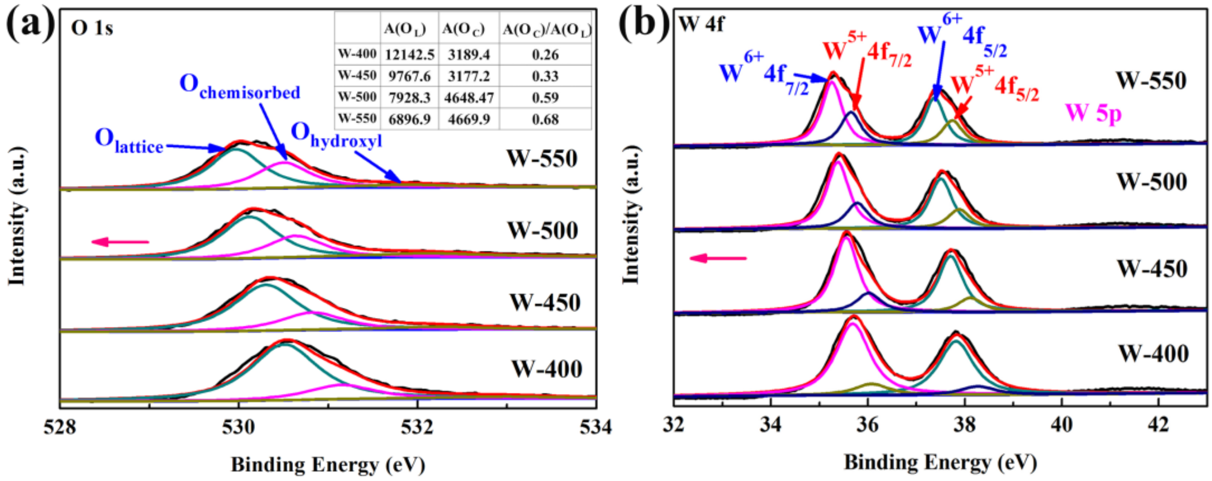

Figure S8. XPS spectra of W-400, W-450, W-500 and W-550: (**a**) O 1s, (**b**) W 4f. (The inset table of a shows the respective peak areas of $O_{lattice}$ and $O_{chemisorbed}$ and the proportion of $O_{chemisorbed}$ / $O_{lattice}$ in W-400, W-450, W-500 and W-550.).

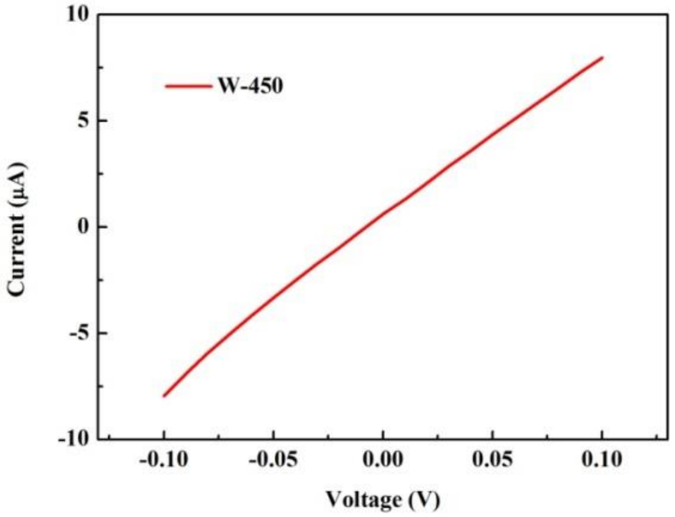

Figure S9. I-V curve of W-450 varying from -0.1V to 0.1V.

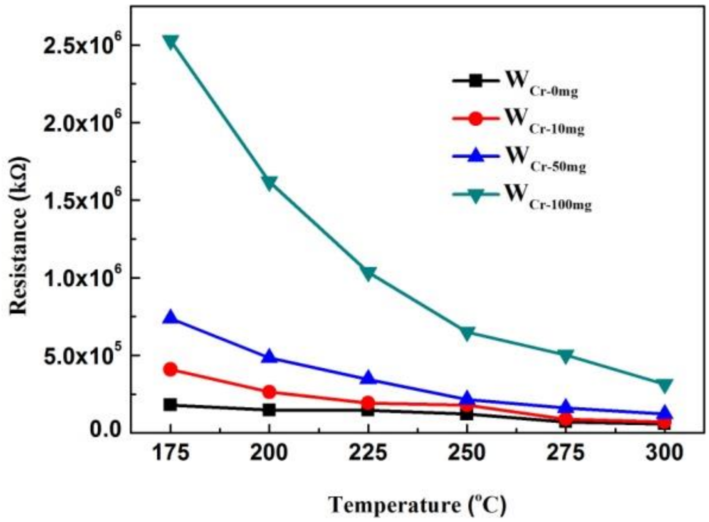

Figure S10. The resistance curve of W_{Cr-0mg}, $W_{Cr-10mg}$, $W_{Cr-50mg}$ and $W_{Cr-100mg}$ at different temperatures.

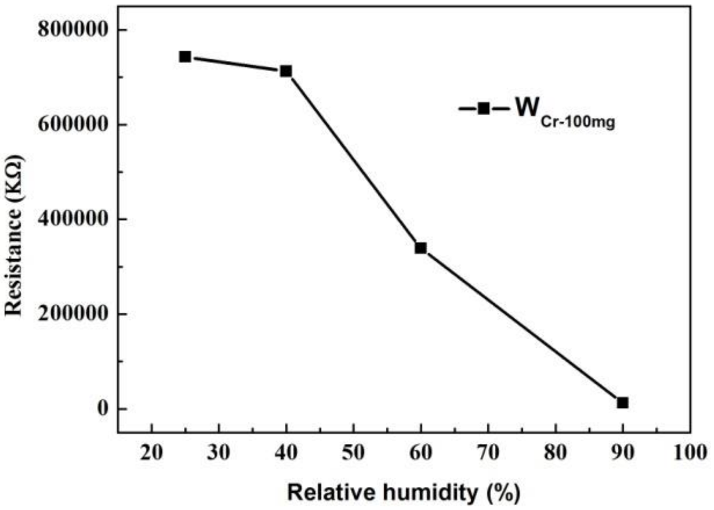

Figure S11. The resistance curve of $W_{Cr-100mg}$ at different humidities.
